# The Relative Timing of Mutations in a Breast Cancer Genome

**DOI:** 10.1371/journal.pone.0064991

**Published:** 2013-06-10

**Authors:** Scott Newman, Karen D. Howarth, Chris D. Greenman, Graham R. Bignell, Simon Tavaré, Paul A. W. Edwards

**Affiliations:** 1 Hutchison/MRC Research Centre and Department of Pathology, University of Cambridge, Cambridge, United Kingdom; 2 Cancer Genome Project, Wellcome Trust Sanger Institute, Wellcome Trust Genome Campus, Cambridge, United Kingdom; 3 CRUK Cambridge Institute, University of Cambridge, Li Ka Shing Centre, Cambridge, United Kingdom; Deutsches Krebsforschungszentrum, Germany

## Abstract

Many tumors have highly rearranged genomes, but a major unknown is the relative importance and timing of genome rearrangements compared to sequence-level mutation. Chromosome instability might arise early, be a late event contributing little to cancer development, or happen as a single catastrophic event. Another unknown is which of the point mutations and rearrangements are selected. To address these questions we show, using the breast cancer cell line HCC1187 as a model, that we can reconstruct the likely history of a breast cancer genome. We assembled probably the most complete map to date of a cancer genome, by combining molecular cytogenetic analysis with sequence data. In particular, we assigned most sequence-level mutations to individual chromosomes by sequencing of flow sorted chromosomes. The parent of origin of each chromosome was assigned from SNP arrays. We were then able to classify most of the mutations as earlier or later according to whether they occurred before or after a landmark event in the evolution of the genome, endoreduplication (duplication of its entire genome). Genome rearrangements and sequence-level mutations were fairly evenly divided earlier and later, suggesting that genetic instability was relatively constant throughout the life of this tumor, and chromosome instability was not a late event. Mutations that caused chromosome instability would be in the earlier set. Strikingly, the great majority of inactivating mutations and in-frame gene fusions happened earlier. The non-random timing of some of the mutations may be evidence that they were selected.

## Introduction

Each individual cancer genome contains an 'archaeological record' of the tumor's history, and recent studies have begun to infer the order in which mutations have occurred [1], [2]. For example, if a certain class of mutations clusters at a certain time in tumor evolution, this might suggest that these mutations were selected at that stage of evolution or that a particular mutation mechanism was active at that time.

Whole genome sequencing studies have begun to uncover hundreds of coding mutations and genome rearrangements in individual tumors but there are only a few frequently mutated genes and many more infrequent mutations [3]. Most mutations seem likely to be passenger events (i.e. random mutations irrelevant to carcinogenesis) and finding the driver events (mutations that give a selective advantage) amongst them represents a considerable challenge [3], [4]. This task is further complicated by a major unknown in cancer biology: the relative importance and timing of genome rearrangements compared to sequence-level mutation. Some suggest chromosome instability might arise early and be essential to tumor suppressor loss [5], [6]. Alternatively, chromosomal instability might be a late event, contributing little to cancer development [7], [8], or might result from a single catastrophic event in some cases [9], [10].

We address the above questions by considering the evolution of the highly mutated genome of HCC1187 [11]. This triple-negative–i.e. ER-negative, PR-negative, *HER2* non-amplified–ductal breast carcinoma cell line is, from a genomics perspective, one of the most intensively studied models of breast cancer as previous studies have examined its genome by whole exome mutation screening, molecular cytogenetics, massively parallel paired end sequencing and transcriptome sequencing [3], [12]–[15].

**Figure 1:**
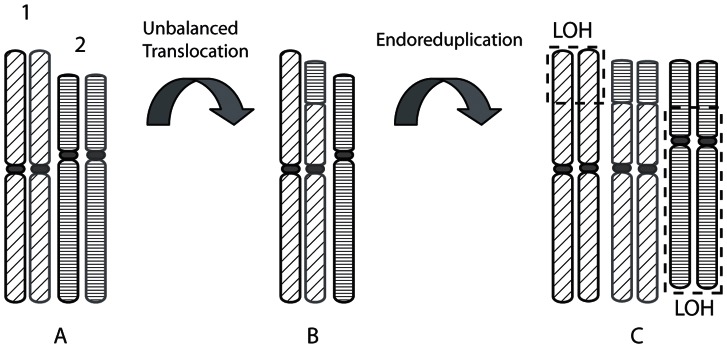
The ‘Monosomic’ pattern of karyotype evolution followed by most breast tumors, with endoreduplication. **A) and B)** In the monosomic pattern of evolution [16], [17], each unbalanced translocation reduces the chromosome number by one, and leaves regions of loss of heterozygosity (LOH). **C)** Often, at some point, endoreduplication occurs, i.e. the whole chromosome complement doubles, to give a duplicated translocation and pairs of chromosome segments showing regions of loss of heterozygosity (dashed boxes). The process may then continue with more unbalanced translocations.

The majority of breast tumors follow a characteristic path of karyotype evolution, in which successive unbalanced chromosome translocations and chromosome losses each reduce the chromosome number by one – termed ‘monosomic evolution’ (Fig. 1) [16], [17]. About one-third of the cancers, like many human cancers, undergo endoreduplication (duplication of the entire karyotype) at some point during this process [17], [18]. As a result, these tumors typically have 65–75 chromosomes at surgery [17]. We show that HCC1187 is one such tumor and we used its endoreduplication as an evolutionary landmark around which we could investigate the timing of different classes of mutation.

## Results

### Mutations in the HCC1187 Genome

We assembled a list of all known sequence-level mutations in the HCC1187 genome (Table S6 in File S2). Non-synonymous coding-sequence mutations were compiled from the coding sequence screen of Wood et al [3] and other, targeted, re-sequencing studies [19]. In total, there are 85 known sequence-level mutations, comprising 75 base substitutions and 10 indels.

Structural mutations, i.e. translocations, inversions, duplications and deletions, and the resulting fused or interrupted genes, were compiled by combining our previous analysis of the karyotype [12], [13] with SNP6 array-CGH copy number data [20] and massively parallel paired-end sequencing [14] (Tables S4, S5 in File S2).

Our previous analysis had identified the breakpoints of chromosome rearrangements larger than about 3 Mb [12], and mapped all balanced breakpoints to gene level, but many unbalanced rearrangements had been mapped only to 1 Mb resolution. SNP6 array data allowed us to map these unbalanced breakpoints more precisely, to around 10 kb resolution, and detect deletions of less than 3 Mb. Paired end sequencing data identified the junctions of around 40 percent of the known rearrangements to sequence level.

Smaller-scale rearrangements, below the resolution of our previous analysis, were also apparent in the SNP6 array data–13 small deletions ranging from 0.26 kb to 2.3 Mb with a median size of 257 kb were predicted. There were also 24 small duplications ranging from 11.7 kb to 2.8 Mb, median size 320 kb. All of these duplications and deletions were absent in the matched normal lymphoblastoid cell line, HCC1187BL. Many of these features were likely to be small interstitial deletions or “head to tail” tandem duplications. Indeed, five of the 13 deletions and 17 of the 24 duplications were confirmed by structural variants detected by the paired-end sequencing [14]. We identified broken genes and possible gene fusions for all these additional structural changes (Tables S1–S6 in File S2). (Paired end sequencing also uncovered further apparent structural variations that were below the resolution of SNP6 segmentation [14]. These were not included in the present analysis, though we checked that they predicted no additional fusion genes).

These structural rearrangements gave rise to at least twelve expressed fusion transcripts, confirmed by RT-PCR and Sanger sequencing: *RGS22-SYCP1*, *CTAGE5-SIP1*, *PLXND1-TMCC1*, *SEC22B-NOTCH2, KLK5-CDH23*, *BC041478-EXOSC10, AGPAT5-MCPH1*, *SUSD1-ROD1/PTBP3*, *SGK1-SLC2A12*, *RHOJ-SYNE2*, *PUM1-TRERF1* and *CTCF-SCUBE2*, some of which have been reported previously ([12]–[15] and Table S4 in File S2). Of these twelve, the first four were predicted to form an in-frame fusion product.

### HCC1187 Endoreduplicated during its History

The HCC1187 karyotype is hypotriploid and highly rearranged, like most breast cancers. The karyotype is highly likely to have evolved via successive chromosome loss, unbalanced translocation and endoreduplication, since this is the predominant pattern in breast tumors (Fig. 1) [17]. We therefore looked for signs of endoreduplication.

**Figure 2:**
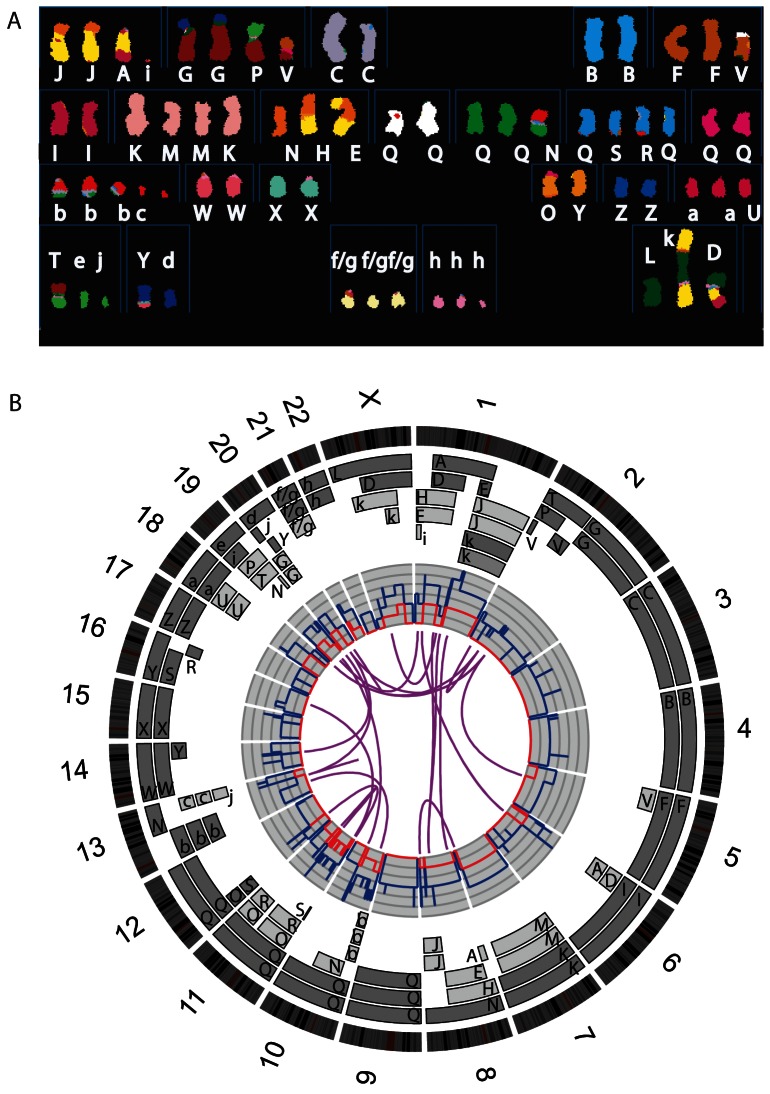
The structure of the HCC1187 genome. **A)** Spectral karyotype as in [44]. Chromosomes are named A-Z and a-k based on their relative sizes as in [12]. Cytogenetic description of the karyotype is in Table S1 in File S2. **B)** Circos plot [45] of the HCC1187 genome: Chromosome ideograms around the outside, oriented clockwise pter to qter. Moving inward, the pale grey and dark grey boxes are chromosome segments observed by array painting [12] with their chromosome of origin indicated. Their parent of origin (light grey and dark grey) was deduced from the number of allelotypes given by PICNIC segmentation (Fig. S1 in File S1). Note that assignment of parents 1 and 2 does not transfer between chromosomes. Dark blue line, total copy number, equivalent to array CGH, from PICNIC. Red line, copy number of the minor allele; where this is zero, the genome is homozygous. Chromosome segments that share a translocation breakpoint were assumed to have the same parental origin. Inner links represent interchromosome translocations identified previously [12]–[14].

The main evidence that endoreduplication had occurred was that a high proportion of the genome had been duplicated precisely once. To make this clearer, we worked out which chromosome segments derived from which parent by analysing how many copies of each genomic segment had the same alleles, using SNP array data (Fig. S1 in File S1). We were able to assign almost all chromosome segments in the karyotype to one or the other allelotype (Fig. 2). This showed that many chromosome segments were present in two copies of the same parental origin, and most of the remainder appear to have evolved from a pair of copies (Fig. 2B). For example, all segments of chromosomes 6 and 7 are present in two copies, while there are two complete copies of chromosome 16 derived from the same parent, one of which has been split by a balanced translocation.

### Inferring the Genome State before Endoreduplication

**Figure 3:**
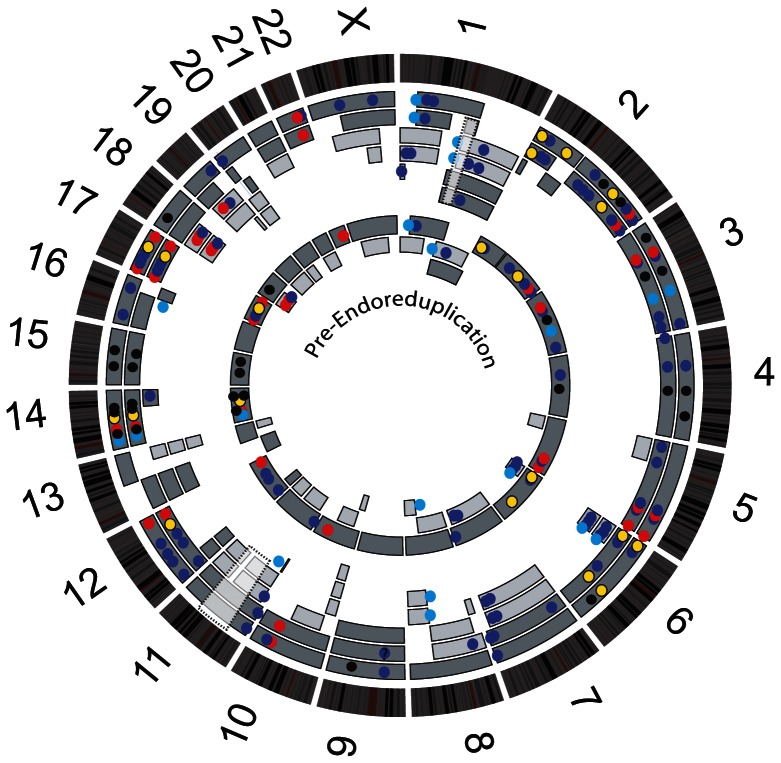
Chromosome segments in HCC1187 and their most probable state before endoreduplication. Chromosome ideograms are drawn around the outside as in Fig. 2. Outer rings are array painting segments as in Fig. 2. Inner rings are chromosome segments that must have been present before endoreduplication. Coloured circles are different types of mutations, on the outer chromosome segment on which they were observed: truncating (red), non-synonymous (blue), small deletion (yellow), small duplication (black), expressed gene fusion (light blue). Mutations that were on two copies of a chromosome segment probably occurred before endoreduplication and are also shown on the inner, pre-endoreduplication genome. Dashed grey boxes on chromosome 1 and 11 indicate regions where parental origin was undetermined, because PICNIC segmentation suggested additional rearrangements had taken place.

Having clear evidence that endoreduplication had occurred in HCC1187, we were able to infer the state of the genome immediately before it doubled (Fig. 3). To do this, we assumed that the simplest possible sequence of events had happened, in particular that endoreduplication accounted for almost all duplications. The resulting picture of the likely history of the karyotype almost exactly fits the suggested monosomic pattern of evolution (Fig. 1), with each unbalanced translocation leading to loss of one chromosome, plus some whole-chromosome losses.

We next determined whether the somatic mutations most likely occurred before or after endoreduplication. As most loci in this genome had duplicated only once we could infer whether a mutation happened before or after endoreduplication: if the mutation occurred before, it would be present in two copies after the duplication, whereas if the mutation occurred after endoreduplication, it would only be present on one of two copies. This classification would be wrong if gene conversion had occurred, i.e. copying of an allele from one chromosome to another, but all of the 83 sequence-level mutations analysed below were found on only one parent of origin, implying that gene conversion was rare or absent in this cell line, as is typical for epithelial cancers [21], [22].

We placed each structural mutation before or after endoreduplication, according to whether the translocation junction or deletion was duplicated, or involved only one copy of a pair of participating chromosomes (Figs. 2 and 3). For the three regions of the genome that were triplicated we assumed that one duplication had occurred at endoreduplication and another had occurred later. We determined the copy number of gene fusions, and confirmed their chromosomal location by fluorescence in situ hybridization (FISH) (not shown). For small deletions and duplications, we determined the copy number of the relevant region relative to flanking regions from array CGH segmentation to assess whether the segment bearing the deletion or duplication had itself been duplicated. Earlier deletions and duplication showed a copy number shift of two or more whereas later events had a copy number shift of only one.

Seven of the twelve fusion transcripts were classified as before endoreduplication; two, *CTCF-SCUBE2* and *BC041478-EXOSC10* were classified later. *AGPAT5-MCPH1* and *SUSD1-ROD1/PTBP3* and *KLK5-CDH23* were undetermined, as their allelic copy number could not be resolved by array CGH or FISH. We were able to place seven deletions earlier, and these were all the homozygous deletions. Five deletions, all heterozygous, were placed later, with one undetermined. We could unambiguously place 14 small duplications relative to endoreduplication: seven earlier and seven later.

**Figure 4:**
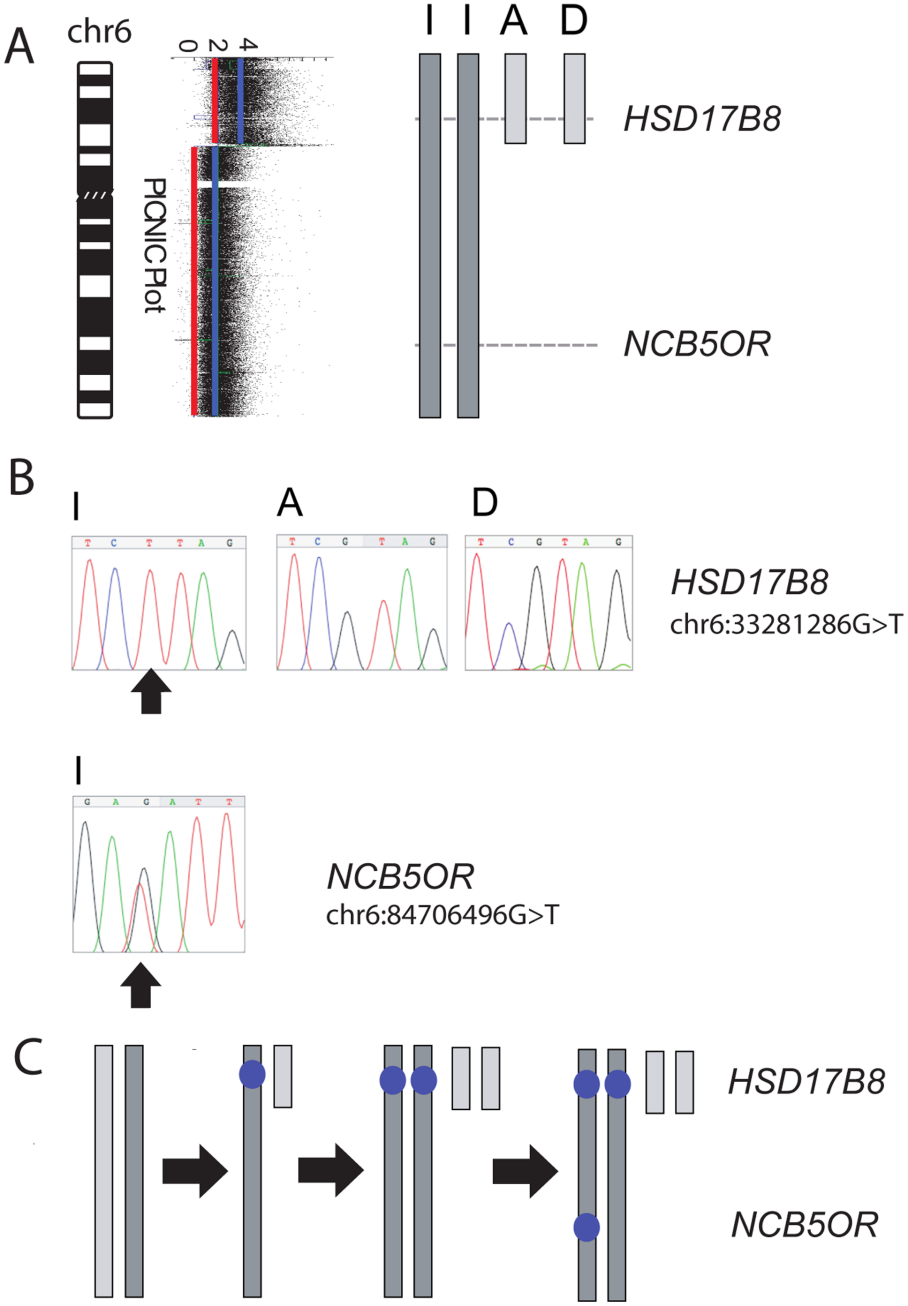
Point mutations on chromosome 6, and whether they occurred before or after endoreduplication. **A)** Deducing the parental origin of chromosome 6 segments: the simplest explanation for the allele combinations (blue and red lines on the aCGH plot) in terms of parental origin. Both copies of chromosome 6 I (chromosome 6 fragments are designated 6 I, 6A, 6D as in ref. [12]) originate from parent 1 and the chromosome 6 segments of 6A and 6D originate from parent 2. Several small copy number steps are omitted for clarity. **B)** Sequence traces show whether mutations are on each isolated chromosome. *HSD17B8*: Chromosome 6I (2 copies) homozygous G>T mutation (black arrow); chromosome 6A and 6D, no mutation. *NCB5OR*: Chromosome 6, heterozygous mutant (black arrow). **C)** The likely evolution of the segments of chromosome 6: unbalanced translocation of one copy of chromosome 6 was followed by duplication of both chromosomes during endoreduplication. *HSD17B8* was mutated on each copy of chromosome 6I, but not on 6A or 6D, while *NCB5OR/CYB5R4* was mutated on only one copy of chromosome 6I. The pre-endoreduplication state was likely to be one normal copy of chromosome 6 with the other having a mutation in *HSD17B8* and having suffered unbalanced translocation. The *NCB5OR/CYB5R4* mutation occurred after endoreduplication.

To assign point mutations to one or two copies of particular chromosomes, we isolated the individual chromosomes in a cell sorter and re-sequenced the mutated exons (Fig. 4). We confirmed this analysis for selected genes by measuring the relative number of mutant and wild-type copies using pyrosequencing (Fig. S2 in File S1). We were able to place 75 of the 85 previously described sequence-level mutations before or after endoreduplication, with only 10 undetermined. Of these ten, two were on a chromosome that was too small to be resolved in flow sorting, and 8 were not possible to score, either because they were found on single-copy genome segments, or they were found in a region where parent of origin could not be determined. Two reported mutations, in *ZNF674* and *HUWE1*, were not found in our sample, therefore presumably occurred in other stocks of the line. They could therefore be classified as later (Fig. 3, Table 1 and Table S6 in File S2).

**Table 1:** Summary of Mutations in HCC1187 and their timing.

	Total Reported	Total Classifiable	Unclassifiable	Earlier	Later	% Earlier
**All Mutations**	144	123	21	61	62	50
**All Structural mutations**	59	48	11	27	21	56
Translocation	22	22	0	13	9	59
Deletion (<2 Mb)	13	12	1	7	5	58
Duplication (<2 Mb)	24	14	10	7	7	50
**All Sequence-level mutations**	85	75	10	34	41	45
Synonymous^a^	4	4	0	0	4	0
Missense	66	58	8	23	35	40
Nonsense	5	5	0	3	2	60
INDEL	10	8	2	8	0	100
**Mutation Classification**						
Nonsense and INDEL mutations	15	13	2	11	2	85
Missense classifiable by SIFT	52	47	5	16	31	34
SIFT non-functional	30	28	2	9	19	32
SIFT Functional	22	19	3	7	12	37
CAN genes	9	9	0	6	3	67
Expressed fusion transcripts	12	9	3	7	2	77
In-frame transcript^b^	4	3	0	3	0	100
Out of Frame transcript^b^	8	6	2	4	2	67

a Few synonymous mutations are known since they were not reported in the main survey of point mutations [3].

b In-frame and out-of-frame expressed fusion transcripts.

### Earlier and Later Mutations

Overall, the proportion of mutations classified as occurring before endoreduplication (earlier) was fairly similar for structural and point mutations (Table 1): 27/48 (56%) of structural changes (translocations, deletions and duplications) and 34/75 (45%) sequence-level changes were classed as earlier (Fig. 3 and Tables S4–S7 in File S2).

Among the structural mutations that could be classified, 13/22 (59%) of chromosome translocations were in the earlier group, while 7/14 (50%) of small duplications were earlier and 7/12 (58%) of small deletions were earlier. For fusion genes, 7/9 (78%) were classified earlier and, interestingly, all three in-frame fusion transcripts that could be classified were classified as earlier.

Of the classifiable sequence-level mutations, 58 were missense mutations, of which 23/58 (40%) fell early. To try to uncover ‘driver’ mutations within this group, we applied the Sorting Intolerant from Tolerant (SIFT) algorithm [23] to all of the point mutations, 47 of which could be scored by this method. Of the missense mutations predicted to be non-functional (tolerated) and so more likely to be random, 9/28 (32%) were earlier, while 7/19 (37%) mutations predicted to be functional (deleterious) were earlier (Table 1, Table S6 in File S2). Wood *et al.* [3] also identified genes likely to be drivers as ‘candidate cancer genes’ (CAN) based on their mutation rate and several other bioinformatic estimates of functionality [3]. The nine CAN genes showed a bias towards the earlier category, six classified earlier (*INHBE, KIAA0427/CTIF, MYH9, PCDHB15, RNU3IP2/RRP9, TP53*) and three in the later category (*ABCB8, KIAA0934/DIP2C, NCB5OR/CYB5R4*).

Strikingly different from the overall distribution of mutations in HCC1187 was the proportion of sequence-level truncation mutations in earlier rather than later categories: All eight classifiable INDEL mutations happened earlier, and combining this figure with nonsense mutations showed 11/13 (85%) protein truncating mutations happened earlier. This difference in proportion (11/13 truncating vs. 23/58 missense) is statistically significant (p<0.01 for chi-squared test with continuity correction).

### Non-random Timing of Mutation Subsets

The distribution of mutations between earlier and later could uncover selective pressure for a mutation to occur at a particular stage in tumor development, or a change in the level of genetic instability. We therefore estimated the number of random and non-randomly timed mutations given the proportions of different mutation classes above.

We used a statistical model to estimate the number of mutations that showed non-random timing. The model assumed that any given class of mutations is a mixture of non-random mutations that must happen earlier (that is, before endoreduplication) and randomly timed mutations that can happen earlier or later. The randomly timed mutations are classified as earlier with probability *p* and later with probability *1-p*, independently for each such mutation. We find the most likely number, *n*, of non-randomly timed mutations (the maximum likelihood estimate, or MLE) and its 95 percent lower confidence bound, given an estimate of *p.* Further details of the model may be found in File S3.

Estimates of *p* based on total missense mutations or those predicted to be non-functional (see Table 1) are 0.40 ( = 23/58) or 0.32 ( = 9/28), respectively, and a plausible upper bound would be 0.59 ( = 13/22), the proportion of earlier chromosome translocations.

Most classes of mutation, including non-synonymous point mutations, chromosome translocations, duplications, deletions, predicted functional mutations and CAN genes did not show any excess of mutation earlier or later. However, the observed proportion of truncating mutations falling earlier (11/13) suggests that *n* >0. When *p*  = 0.4, the MLE is *n* = 10 mutations that had to happen before endoreduplication, with a lower confidence bound of 6 (File S3) [24]. For *p* = 0.32 *n* = 10, lower bound 7. Thus our simple statistical model suggests that a number of the truncating mutations had to occur before endoreduplication.

When we use the high estimate for *p, p* = 0.59, the MLE was *n = *9, but the lower confidence bound is 0, so data from more tumors would be required.

## Discussion

We present one of the most complete studies of any cancer genome to date, combining the coding sequence scan of Wood et al [3] with molecular cytogenetic analysis of genome rearrangement. We were able to deduce for most of the mutations and genome rearrangements whether they most likely occurred before or after endoreduplication of the genome, giving us a picture of the pattern of mutation before and after this time point, for this case. Such detailed analysis was limited to a single cell line as this was the only example so far of a breast cancer cell line for which there is rather complete coding sequence data, cytogenetic data and evidence of endoreduplication, but it serves to demonstrate the feasibility and potential interest of the approach.

### The Earlier Versus Later Classification

Endoreduplication in HCC1187 proved to be a useful milestone, because numbers of structural changes and point mutations were fairly equally distributed between the earlier and later categories, implying that endoreduplication occurred 32–60% of the way through the mutational history of this genome.

Interpretation of mutation timing depends on the accuracy of our earlier and later classification of mutations. We were confident that the tumor had undergone endoreduplication as it showed two characteristic signatures of this phenomenon: multiple duplicated rearrangements and multiple duplicated homozygous regions (Fig. 2). Given that there had been an endoreduplication, we reconstructed the main steps of HCC1187 karyotype evolution by assuming that the simplest possible sequence of events had happened. Implicit was the assumption that, as far as possible, all duplications had occurred at endoreduplication. The deduced sequence of chromosome changes (Fig. 3) was consistent with monosomic evolution (Fig. 1). Three duplications could not be explained by endoreduplication: these were three chromosome segments of the same parental origin that were present in three copies. The simplest route to these triplications was via endoreduplication followed by an additional single-chromosome duplication.

A few steps in the evolution may have been more complex, but this would not have altered the earlier versus later classification very often. Specifically, if all three triplicated chromosomes had taken a more complex evolutionary route (perhaps duplication followed by endoreduplication, followed by loss), the classification of no more than three point mutations could be affected, moving them from the later category to the ‘undetermined’ class.

Some mutations were omitted from analysis. These were from the complex regions of 10 p and 11 q where the parent of origin could not be determined. The omitted mutations comprised eight non-synonymous missense and two truncating mutations. Even if we consider the most unfavorable case, that the two truncating mutations were classified as later, the MLE for the number of non-random truncation mutations in the earlier group is is *n*  = 9, with a 95% lower bound of 3 when *p*  = 0.4, and *n*  = 5 or 6 with a 95% lower bound of 0 when *p*  = 0.59.

### Timing of Genetic Instability and Other Functions

This earlier versus later classification may help us to understand a variety of issues including the timing and origins of chromosome instability and the drivers versus passengers problem for this particular tumor.

There has been much discussion of when chromosomal instability occurs, for example some have suggested it as a key facilitator of early tumorigenesis, notably causing loss of heterozygosity of APC in colorectal cancers [21]. In contrast, some suggest that the extensive rearrangements of carcinoma karyotypes might be late progression events [7], [8]. Others favor a transient period of chromosome instability, either at ‘crisis’ caused by telomere loss, or at some other catastrophic event such as ‘chromothripsis’, in which one or more chromosomes undergo massive rearrangement, apparently in a single event [9], [10].

We observed roughly equal ratios of structural to sequence-level mutations earlier and later. Although there are other possibilities, a reasonable explanation is that both mutational processes were happening at approximately the same rate for much of the evolution of the tumour. We did not see, for example, a much higher ratio of rearrangements to point mutations in the earlier class, as expected if most of the rearrangements had occurred during a telomere crisis before endoreduplication.

An important value of the classification is that mutations that may cause ongoing chromosome instability must generally be in the ‘earlier’ group as, by definition, they must pre-date almost all chromosome changes, which were quite numerous before endoreduplication. Among the mutated genes that might contribute to chromosome instability were *TP53*, *BAP1* and *PAXIP1*, and all were indeed classified as earlier. BAP1/UCHL2 (*BRCA1*-associated protein1/ubiquitin carboxy-terminal hydrolase 2) was discovered as a binding partner of BRCA1 and appears to participate in the DNA damage response by interacting with BRCA1 and BARD1 [25]; [26]. It is a deubiquitinase that controls ubiquitination of Histone H2A, and is also a component of the Polycomb repressive deubiquitinase (PR-DUB) complex. Inactivating mutations of *BAP1* have been found particularly in uveal melanomas [27], and as germline mutations that predispose to melanoma [28] and other cancers. Based on this, Stephens *et al.* [29] identified *BAP1* mutations as likely driver mutations in breast cancer. PAXIP1 also seems to be part of the DNA damage signalling system, interacting through its multiple BRCT domains [30]. (Another gene relevant to genetic instability, but at the sequence level, was *HUWE1*, but we did not find the reported *HUWE1* mutation in our stock. HUWE1 is implicated in base excision repair according to the UniProt database).

We also considered how other functions relevant to breast cancer might relate to timing, using the Entrez and Gene Ontology databases (Table S7 in File S2). Although we do not know which mutations were driver mutations, a gene involved in steroid hormone synthesis, *HSD17B8*, was mutated earlier, while genes encoding covalent modifiers of histones (*HUWE1*, *IPO7*, *MLL4*, *PAXIP1*, *PRKAA2*) were, except for *PAXIP1*, mutated later. Some of these histone modifiers might affect differentiation, while PAXIP1 might affect genome stability. However, other functional groupings were less informative: 17 of the genes mutated in HCC1187 were associated with actin and the cortical cytoskeleton or integrin signalling–functions highlighted in a study of triple-negative breast cancers [31] (HCC1187 is triple-negative)–but they were fairly evenly divided between earlier and later. Similarly the many genes associated with G-protein coupled receptors and Rho signalling, and genes associated with apoptosis, were fairly evenly divided.

### Timing and Evidence for Selection

A non-random distribution of mutations between earlier and later categories could be evidence that a substantial number of mutations were drivers rather than passengers. The group of mutations showing greatest deviation from the typical pattern of around 40% earlier was the truncating mutations (and especially indels). A non-random distribution could be explained in two ways: i) the rate of indel mutations was high before endoreduplication and low after, relative to most other types of mutation ii) passenger indels accumulated in the same way as other passenger mutations but more indels accumulated early because they were selected. We consider (ii) most likely because for 9/11 earlier truncating mutations, a chromosome loss before endoreduplication caused loss of the second wild type allele. This is consistent with chromosome instability facilitating early tumor suppressor loss, as has been suggested previously [21]. Indeed, the earlier truncation mutations include known and candidate tumor suppressor genes *TP53, BAP1* (*BRCA1*-associated protein), *CTNNA1* (CateninA1) and *NFKBIA* (nuclear factor of kappa light polypeptide gene enhancer in B-cells inhibitor) [20], [29], [32]–[34]; others were *AVPI1*, *GMCL1L/GMCL1P1*, *GPR81HCAR*, *MYH9*, *SLC4A3*, *ELP2* and *TRIM47*.

These data, therefore, support the view that early tumor suppressor loss is consistent with tumor evolving monosomically and that driver mutations that cause gene inactivation will be concentrated pre-endoreduplication. An explanation for this phenomenon is that loss or inactivation of two alleles pre-endoreduplication is more likely than loss/inactivation of four alleles post-endoreduplication [16], [17]. Gain of function mutations are not under the same numerical constraints as tumor suppressors. Where two hits are required to impair tumor suppressor gene function, only a single mutation is required for oncogenic gains of function and we might, therefore, see these mutations either side of endoreduplication. Fusion genes–whose importance in the common epithelial cancers has only recently been acknowledged [35], [36]– were formed throughout the evolution of this tumor but, interestingly, the in-frame fusions (most likely to be translated into functional proteins) were all formed early. Although the numbers are admittedly too small for statistical certainty, this makes the early in-frame gene fusions also good candidates for selected events.

Interpretations of the earlier and later classes depend on when HCC1187 endoreduplicated. There is some evidence that endoreduplication occurred *in vivo* in this case. The original ploidy of HCC1187 was not reported, only that shortly after its derivation, HCC1187 had multiple ploidy indices by flow cytometry [37]. However, around 60 percent of mutations occurred after endoreduplication. It would be surprising if so many happened in culture, given that cell lines largely recapitulate the genomic aberrations observed in primary tumors [38]. If endoreduplication happened *in vitro*, only ‘earlier’ mutations happened *in vivo,* and all driver mutations will be in the ‘earlier’ set, whereas if endoreduplication happened *in vivo* (as is often the case in breast tumours [17]), some driving mutations will be present in the ‘later’ group. In either case our estimate of non-randomly timed mutations remains the same.

### Comparison with Mutations in Breast Cancers

To attempt to identify mutations in HCC1187 that are recurrent or known drivers, and relate this to timing, we compared sequencing data from breast tumours, both genomic sequencing data [29], [31], [39]–[41] and fusion gene data from transcriptome sequencing [15], [42]. However, this was largely uninformative, because of the heterogeneity and variability of mutations among cancer cases, and the still limited amount of data available. For example, Stephens et al [29] identified 31 genes as targets of driver mutations in breast cancers, from sequencing of 100 exomes and comparison with known drivers in other cancers, but the individual tumours had an average of only 1.7 of these genes mutated, 1.3 if *TP53* is excluded. Unsurprisingly, then, in HCC1187 only two of these drivers, *TP53* and *BAP1*, were mutated (both earlier). Similarly, of the genes considered likely drivers by Shah et al [31] in triple negative breast cancers, only *SYNE2* and *TP53* were mutated in HCC1187–*SYNE2* is fused, also earlier. Nevertheless, many other genes mutated in HCC1187 are mutated or rearranged occasionally in the datasets above, so may be recurrent at a modest level (Tables S4, S7 in File S2). Several genes involved in fusions have been reported to be fused or rearranged in other cases–*AGPAT5*, *NOTCH2*, *PUM1*, *SEC22B*, *SGK1* and *TRERF1* (all early or unclassified), while several are mutated at sequence level, notably *SYNE2*. Among the genes in homozygous deletions (all earlier) with four or more reported mutations or rearrangements were *SCN1A*, *FBXL20* and *MYO9A*; while among the point-mutated genes in HCC1187, apart from the known drivers *BAP1* and *TP53*, ones with four or more reported mutations or rearrangements were *CAMTA1*, *ITIH6*/*ITIH5L*, *LHCGR*, *SPEN*, *TP53* and *ZNF142* (3 of which were earlier, 3 later). Many of the other genes have been found mutated in cancers other than breast, including *CTNNA1* and *NFKBIA* (both earlier, see above). Others have cancer-relevant functions, such as steroid hormone synthesis (*HSD17B8*, earlier), and covalent modification of histones (*HUWE1*, *IPO7*, *MLL4*, *PAXIP1*, *PRKAA2*, all later except *PAXIP1*) (Table S7 in File S2).

### Applicability to Sequencing Data

Our theoretical framework and statistical methods could be applied, in a modified form, to sequencing data from other endoreduplicated cell lines and primary tumours, indeed the idea of placing mutations before or after a duplication event has already been exploited [1], [18]. Endoreduplication is a common process in epithelial cancers, estimated to occur in more than 50% of breast cancers [17], [18]. Endoreduplicated genomes can often be identifed by copy number and allele ratios [18], for example, a large proportion of a recently-endoreduplicated genome will often be present either in four copies and heterozygous, or two homozygous copies (Fig. 4). We relied on flow sorting of chromosomes to quantify our mutations, but the proportion of mutant and reference alleles could be deduced, for example, by counting reads from deep massively-parallel sequencing. Earlier mutations will usually be homozygous in diploid regions, or account for approximately 50% of mutant reads in tetraploid regions. Distinguishing between earlier and later events in large datasets may help identify genes or pathways that must be mutated earlier or later in a given tumour type.

### Conclusion

In conclusion, we provide evidence that, in this cell line, chromosome instability and rearrangement was not a late and irrelevant event, and that the great majority of inactivating mutations and expressed gene fusions appear to have happened early, and this suggests that most of them were selected.

## Materials and Methods

Cell line HCC1187 was from ATCC and was grown in RPMI 1640 medium containing 10% foetal calf serum. Metaphase preparations and flow sorting of chromosomes were as described previously [12]. Flow sorted chromosomes were amplified by Genomiphi whole genome amplification (GE Healthcare, Bucks, UK). All flow sorted chromosome fractions were hybridized to normal metaphases to confirm that they were substantially pure (not shown).

For sequencing, exons with flanking intronic sequence were amplified using published primer sequences [3]. Reactions were performed as above using 25 ng flow-sorted and amplified chromosomes or HCC1187 whole genomic DNA as a target. PCR products were cleaned up using Nucleofast 96 PCR cleanup kit (Clontech, Mountain View, CA) and sequenced in both directions using the same primers as for amplification with BigDye v3.1 (Applied Biosystems, Foster City, CA) according to manufacturer’s instructions on an ABI 3700 capillary DNA sequencer.

SNP6 data [20] are available online (www.sanger.ac.uk/cgi-bin/genetics/CGP). Data were viewed as PICNIC-segmented graphical output [43].

## Supporting Information

File S1 Figures S1 and S2 Figures S1 and S2 are provided in a single pdf document. Figure S1. Segmentation by PICNIC algorithm reveals ‘Parent A’ and ‘Parent B’ origin of segments of chromosome 13. Figure S2. Pyrosequencing confirmation of the *HSD17B8* mutation.
(PDF)

File S2 Tables S1–S7 Tables provided as a single spreadsheet in Excel format. Table S1, cytogenetic descriptions of genome rearrangements in HCC1187, from ref. 12. Table S2, array-CGH data segmented PICNIC algorithm. Table S3, genome segments originally identified by array painting in ref. 12, with breakpoints refined by comparison with array CGH data in table S2. Table S4, Expressed Fusion Genes. Table S5, Deletions and duplications of less than 2 Mb, identified from array CGH. Table S6, Sequence-level mutations, with comments and annotations as described in the text. Table S7, all genes affected by mutation, with timing, recurrence of mutation in breast cancer, and brief gene annotation.
(XLS)

File S3 **Details of statistical model.**
(PDF)

## References

[1] Durinck S, Ho C, Wang NJ, Liao W, Jakkula LR, et al (2011) Temporal dissection of tumorigenesis in primary cancers. Cancer Discovery 1: 137–143. 21984974 10.1158/2159-8290.CD-11-0028 PMC3187561

[2] Greenman C, Pleasance E, Newman S, Yang F, Fu B, et al (2012) Estimation of rearrangement phylogeny for cancer genomes. Genome Res 22: 346–361. 21994251 10.1101/gr.118414.110 PMC3266042

[3] Wood LD, Parsons DW, Jones S, Lin J, Sjoblom T, et al.. (2007). The Genomic Landscapes of Human Breast and Colorectal Cancers. Science 318, 1108–1113. 10.1126/science.1145720 17932254

[4] Greenman C, Stephens P, Smith R, Dalgliesh GL, Hunter C, et al (2007) Patterns of somatic mutation in human cancer genomes. Nature 446: 153–158. 17344846 10.1038/nature05610 PMC2712719

[5] Nowak MA, Komarova NL, Sengupta A, Jallepalli PV, Shih I-M, et al (2002) The role of chromosomal instability in tumor initiation. Proc Natl Acad Sci USA 99: 16226–16231. 12446840 10.1073/pnas.202617399 PMC138593

[6] Rajagopalan H, Nowak MA, Vogelstein B, Lengauer C (2003) The significance of unstable chromosomes in colorectal cancer. Nat Rev Cancer 3: 695–701. 12951588 10.1038/nrc1165

[7] Johansson B, Mertens F, Mitelman F (1996) Primary vs. secondary neoplasia-associated chromosomal abnormalities - balanced rearrangements vs. genomic imbalances? Genes Chromosomes Cancer 16: 155–163. 8814447 10.1002/(SICI)1098-2264(199607)16:3<155::AID-GCC1>3.0.CO;2-Y

[8] Sieber OM, Heinimann K, Tomlinson IPM (2003) Genomic instability–the engine of tumorigenesis? Nat Rev Cancer 3: 701–708. 12951589 10.1038/nrc1170

[9] Chin K, de Solorzano CO, Knowles D, Jones A, Chou W, et al (2004) In situ analyses of genome instability in breast cancer. Nat Genet 36: 984–988. 15300252 10.1038/ng1409

[10] Stephens PJ, Greenman CD, Fu B, Yang F, Bignell GR, et al (2011) Massive genomic rearrangement acquired in a single catastrophic event during cancer development. Cell 144: 27–40. 21215367 10.1016/j.cell.2010.11.055 PMC3065307

[11] Gazdar AF, Kurvari V, Virmani A, Gollahon L, Sakaguchi M, et al (1998) Characterization of paired tumor and non-tumor cell lines established from patients with breast cancer. Int J Cancer 78: 766–774. 9833771 10.1002/(sici)1097-0215(19981209)78:6<766::aid-ijc15>3.0.co;2-l

[12] Howarth KD, Blood KA, Ng BL, Beavis JC, Chua Y, et al (2008) Array painting reveals a high frequency of balanced translocations in breast cancer cell lines that break in cancer-relevant genes. Oncogene 27: 3345. 18084325 10.1038/sj.onc.1210993 PMC2423006

[13] Howarth KD, Pole JC, Beavis JC, Batty EM, Newman S, et al (2011) Large duplications at reciprocal translocation breakpoints that might be the counterpart of large deletions and could arise from stalled replication bubbles. Genome Res 21: 525–534. 21252201 10.1101/gr.114116.110 PMC3065700

[14] Stephens PJ, McBride DJ, Lin M-L, Varela I, Pleasance ED, et al (2009) Complex landscapes of somatic rearrangement in human breast cancer genomes. Nature 462: 1005–1010. 20033038 10.1038/nature08645 PMC3398135

[15] Robinson DR, Kalyana-Sundaram S, Wu Y-M, Shankar S, Cao X, et al (2011) Functionally recurrent rearrangements of the MAST kinase and Notch gene families in breast cancer. Nat Med 17: 1646–51. 22101766 10.1038/nm.2580 PMC3233654

[16] Muleris M, Salmon RJ, Dutrillaux B (1988) Existence of two distinct processes of chromosomal evolution in near-diploid colorectal tumors. Cancer Genet Cytogenet 32: 43–50. 3162707 10.1016/0165-4608(88)90310-x

[17] Dutrillaux B, Gerbault-Seureau M, Remvikos Y, Zafrani B, Prieur M (1991) Breast cancer genetic evolution: I. Data from cytogenetics and DNA content. Breast Cancer Res Treat 19: 245–55. 1663804 10.1007/BF01961161

[18] Carter SL, Cibulskis K, Helman E, McKenna A, Shen H, et al (2012) Absolute quantification of somatic DNA alterations in human cancer. Nat Biotechnol. 30: 413–21. 10.1038/nbt.2203 PMC4383288 22544022

[19] Forbes SA, Tang G, Bindal N, Bamford S, Dawson E, et al (2010) COSMIC (the Catalogue of Somatic Mutations in Cancer): a resource to investigate acquired mutations in human cancer. Nucleic Acids Res 38: D652–657. 19906727 10.1093/nar/gkp995 PMC2808858

[20] Bignell GR, Greenman CD, Davies H, Butler AP, Edkins S, et al (2010) Signatures of mutation and selection in the cancer genome. Nature 463: 893–898. 20164919 10.1038/nature08768 PMC3145113

[21] Thiagalingam S, Laken S, Willson JK, Markowitz SD, Kinzler KW, et al.. (2001). Mechanisms underlying losses of heterozygosity in human colorectal Cancers. Proc. Natl. Acad. Sci. U.S.A 98, 2698–2702. 10.1073/pnas.051625398 PMC30201 11226302

[22] Ogiwara H, Kohno T, Nakanishi H, Nagayama K, Sato M, et al (2008) Unbalanced translocation, a major chromosome alteration causing loss of heterozygosity in human lung cancer. Oncogene 27: 4788–4797. 18408757 10.1038/onc.2008.113

[23] Ng PC, Henikoff S (2003) SIFT: Predicting amino acid changes that affect protein function. Nucleic Acids Res 31: 3812–3814. 12824425 10.1093/nar/gkg509 PMC168916

[24] Tingley M, Li C (1993) A note on obtaining confidence intervals for discrete parameters. The American Statistician 47: 20–23.

[25] Jensen DE, Proctor M, Marquis ST, Gardner HP, Ha SI, et al (1998) BAP1: a novel ubiquitin hydrolase which binds to the BRCA1 RING finger and enhances BRCA1-mediated cell growth suppression. Oncogene. 16: 1097–112. 10.1038/sj.onc.1201861 9528852

[26] Nishikawa H, Wu W, Koike A, Kojima R, Gomi H, et al (2009) BRCA1-associated protein 1 interferes with BRCA1/BARD1 RING heterodimer activity. Cancer Res. 69: 111–9. 10.1158/0008-5472.CAN-08-3355 19117993

[27] Harbour JW, Onken MD, Roberson EDO, Duan S, Cao L, et al.. (2010). Frequent Mutation of BAP1 in Metastasizing Uveal Melanomas. Science 330, 1410–1413. 10.1126/science.1194472 PMC3087380 21051595

[28] Wiesner T, Obenauf AC, Murali R, Fried I, Griewank KG, et al (2011) Germline mutations in BAP1 predispose to melanocytic tumors. Nat Genet. 43: 1018–21. 10.1038/ng.910 PMC3328403 21874003

[29] Stephens PJ, Tarpey PS, Davies H, Van Loo P, Greenman C, et al (2012) The landscape of cancer genes and mutational processes in breast cancer. Nature. 486: 400–4. 10.1038/nature11017 PMC3428862 22722201

[30] Woods NT, Mesquita RD, Sweet M, Carvalho MA, Li X, et al (2012) Charting the landscape of tandem BRCT domain-mediated protein interactions. Sci Signal. 5: rs6. 10.1126/scisignal.2002255 PMC4064718 22990118

[31] Shah SP, Roth A, Goya R, Oloumi A, Ha G, et al (2012) The clonal and mutational evolution spectrum of primary triple-negative breast cancers. Nature 486: 395–9. 22495314 10.1038/nature10933 PMC3863681

[32] Osborne J, Lake A, Alexander FE, Taylor GM, Jarrett RF (2005) Germline mutations and polymorphisms in the NFKBIA gene in Hodgkin lymphoma. Int J Cancer 116: 646–651. 15858823 10.1002/ijc.21036

[33] Ding L, Ellis MJ, Li S, Larson DE, Chen K, et al (2010) Genome remodelling in a basal-like breast cancer metastasis and xenograft. Nature 464: 999–1005. 20393555 10.1038/nature08989 PMC2872544

[34] Liu X, Yu H, Yang W, Zhou X, Lu H, et al (2010) Mutations of NFKBIA in biopsy specimens from Hodgkin lymphoma. Cancer Genet Cytogenet 197: 152–157. 20193848 10.1016/j.cancergencyto.2009.11.005

[35] Edwards PAW (2010) Fusion genes and chromosome translocations in the common epithelial cancers. J Pathol. 220: 244–54. 10.1002/path.2632 19921709

[36] Edwards PAW, Howarth KD (2012) Are breast cancers driven by fusion genes? Breast Cancer Res 14: 303. 10.1186/bcr3122 PMC3446366 22424054

[37] Wistuba II, Behrens C, Milchgrub S, Syed S, Ahmadian M, et al.. (1998). Comparison of features of human breast cancer cell lines and their corresponding tumors. Clin. Cancer Res 4, 2931–2938. 9865903

[38] Neve RM, Chin K, Fridlyand J, Yeh J, Baehner FL, et al.. (2006). A collection of breast cancer cell lines for the study of functionally distinct cancer subtypes. Cancer Cell 10, 515–527. 10.1016/j.ccr.2006.10.008 PMC2730521 17157791

[39] Nik-Zainal S, Alexandrov LB, Wedge DC, Van Loo P, Greenman CD, et al (2012) Mutational Processes Molding the Genomes of 21 Breast Cancers. Cell. 149: 979–93. 10.1016/j.cell.2012.04.024 PMC3414841 22608084

[40] Ha G, Roth A, Lai D, Bashashati A, Ding J, et al (2012) Integrative analysis of genome-wide loss of heterozygosity and mono-allelic expression at nucleotide resolution reveals disrupted pathways in triple negative breast cancer. Genome Res 10: 1995–2007. 10.1101/gr.137570.112 PMC3460194 22637570

[41] Banerji S, Cibulskis K, Rangel-Escareño C, Brown KK, Carter SL, et al (2012) Sequence analysis of mutations and translocations across breast cancer subtypes. Nature 486: 405–9. 22722202 10.1038/nature11154 PMC4148686

[42] Kim D, Salzberg SL (2011) TopHat-Fusion: an algorithm for discovery of novel fusion transcripts. Genome Biol. 12: R72. 10.1186/gb-2011-12-8-r72 PMC3245612 21835007

[43] Greenman CD, Bignell G, Butler A, Edkins S, Hinton J, et al.. (2010). PICNIC: an algorithm to predict absolute allelic copy number variation with microarray cancer data. Biostatistics 11, 164–175. 10.1093/biostatistics/kxp045 PMC2800165 19837654

[44] Davidson JM, Gorringe KL, Chin SF, Orsetti B, Besret C, et al.. (2000). Molecular cytogenetic analysis of breast cancer cell lines. Br J Cancer 83, 1309–1317. 10.1054/bjoc.2000.1458 PMC2408781 11044355

[45] Krzywinski M, Schein J, Birol I, Connors J, Gascoyne R, et al (2009) Circos: an information aesthetic for comparative genomics. Genome Res 19: 1639–1645. 19541911 10.1101/gr.092759.109 PMC2752132

