# Supplementary material for: The Relative Timing of Mutations in a Breast Cancer Genome
Source: PLoS One. 2013 Jun 10;8(6):e64991. doi: 10.1371/journal.pone.0064991 (PMC3677865; doi:10.1371/journal.pone.0064991)
Supplement: File S1 Figures S1 and S2 — Figures S1 and S2 are provided in a single pdf document. Figure S1. Segmentation by PICNIC algorithm reveals ‘Parent A’ and ‘Parent B’ origin of segments of chromosome 13. Figure S2. Pyrosequencing confirmation of the HSD17B8 mutation. (PDF) [file pone.0064991.s001.pdf]

Figure S1

Assignment of arbitrary parental origin of chromosome segments.

PICNIC array segmentation algorithm identifies the overall copy number – similar to standard array CGH, but also the copy number of the minor allele. For example, in a normal diploid region the total copy number is two, but the “minor allele” copy number is one. In a region of loss of heterozygosity the minor allele copy number must, by definition, be zero.

We combined chromosome segments from array painting (12), their shared breakpoints and PICNIC zygosity data, and were able to assign an arbitrary ‘Parent A’ or ‘Parent B’ origin to virtually all chromosome segments, using the fact that shared breakpoints or shared alleles between two loci imply they had a common ancestor. Note that the parent of origin inference does not apply between chromosomes. For example in Fig 2 we cannot say if chromosomes three and four have the same parent of origin or different.

continued overleaf...

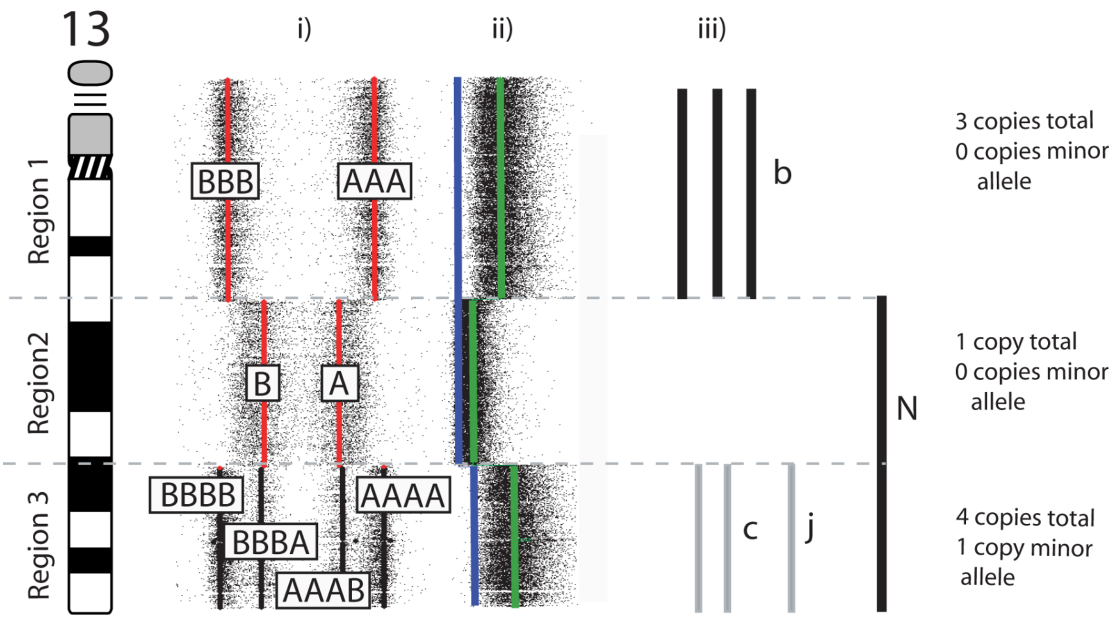

Figure S1. Segmentation by PICNIC algorithm reveals 'Parent A' and 'Parent B' origin of segments of chromosome 13. i) Segmentation by PICNIC algorithm. 'PICNIC plot' gives zygosity, i.e. parental origins, by plotting SNP calls AA, AB, ABB etc. Equal representation of both SNP alleles, i.e. AB, AABB, etc, would be plotted on the centreline. Pure A calls are to the right, with AA and AAA further out, and pure B to the left. Mixed calls of AAB, AAAB, etc fall between corresponding pure A calls and the centreline. Red lines indicates regions of homozygosity. ii) Total segmented copy number (green), equivalent to CGH, plotted left to right and minor allele copy number (blue line). iii) Segments of chromosome identified by array painting as chromosome b, c, j and N with their inferred parentA/parentB origin (A=black, B=grey). In **Region 1**, Total copy number is three, and region is homozygous, since there are only two combinations of alleles, pure A and pure B. Therefore the three copies of chromosome b share the same arbitrary parental origin. In **Region 2**, there is one copy, homozygous. The Chromosome 13 segments in chromosomes b and N are likely to be products of an ancestral translocation, since their breakpoints are the same to 6kb resolution and they add up to a complete chromosome 13. This means that originally the b and N segments were joined so they must have belonged to the same chromosome. **Region 3**: Four copies with four allele combinations indicating that three copies are from the same parent. Peaks c and j share breakpoints, so are derived from the same chromosome, and must be of different parental origin from peak N to account for the allele combinations. In Conclusion, chromosomes b and N are from one parent, while chromosomes c and j are from the other parent.

Figure S2

Confirmation by pyrosequencing

To confirm that the proportion of mutant and non-mutant copies of each gene in the isolated chromosome preparation was accurate, pyrosequencing was used on selected examples. Seven of the point mutations, in CD2, FLJ20422, GPNMB, HSD17B8, ITIH5L, KIAA0427 and MLL4, were investigated. Pyrosequencing assays were designed as for SNP quantification using Pyrosequencing Assay Design Software v 1.0 software (Biotage, Charlotte, NC). Pyrosequencing PCR was carried out using a Pyrosequencer PSQ 96MA, (Biotage) PyroMark Gold reagents (Biotage), steptavidin-sepharose beads (GE Healthcare), microtitre plate (Biotage), PyroMark Vacuum Prep Workstation (Biotage) as per manufacturer's instructions.

In each case, the ratio of normal to mutant alleles found by Sanger sequencing of flow sorted chromosomes was confirmed by pyroseqencing of whole HCC1187 genomic DNA. For example the mutant form of the HSD17B8 gene was shown by Sanger sequencing to be present on both normal copies of chromosome 6 and absent from both translocated chromosome 6 copies. Pyrosequencing showed a 50:50 normal to mutant ratio in whole genomic DNA, as expected. Furthermore, pyrosequencing of flow sorted chromosomes confirmed the 0% mutant in the translocated chromosome 6 copies and 100% mutant in the untranslocated chromosome 6 copies. Thus non-specific genomic contamination of flow sorted chromosomes, if present at all, was at a level too low to detect by either Sanger sequencing or pyrosequencing.

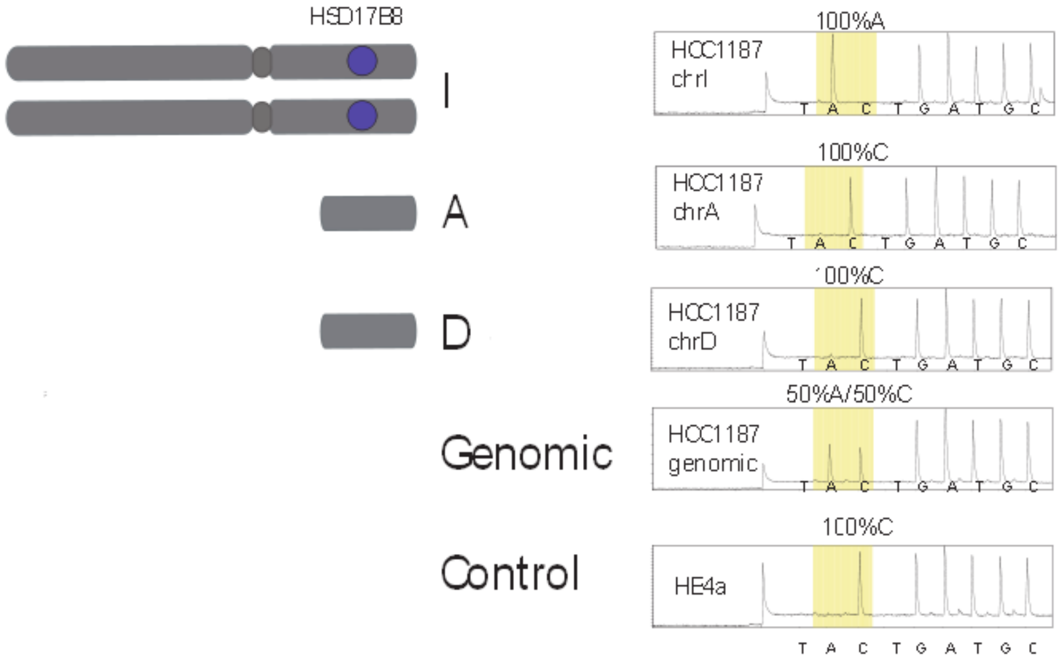

Supplemental Figure 2. Pyrosequencing confirmation of the *HSD17B8* mutation. Left images are chromosome 6 segments I,A and D. The pyrosequencing assay used the reverse strand so the *HSD17B8* G>T mutation would appear to be a C>A. Right hand boxes are quantitative "pyrograms." Control genomic DNA showed 0% mutant bases whereas HCC1187 showed 50% mutant:wild type. Chromosome I showed 100% mutant alleles. As there are two copies of chromosome I in HCC1187 we can conclude that the mutation is *homozygous* with respect to chromosome I. The mutation was not found on chromosomes A and D. Pyrosequencing confirmed the pattern of mutations observed by Sanger sequencing (Fig 4).
