# Supplementary material for: The Relative Timing of Mutations in a Breast Cancer Genome
Source: PLoS One. 2013 Jun 10;8(6):e64991. doi: 10.1371/journal.pone.0064991 (PMC3677865; doi:10.1371/journal.pone.0064991)
Supplement: File S3 — Details of statistical model. (PDF) [file pone.0064991.s003.pdf]

Statistical estimates of the number of non-randomly distributed mutations.

Suppose that $X_1, X_2, \ldots, X_m$ is an independent sample of size m, each taking values in $\{0, 1\}$. Of the m observations, an unknown number n take the value 1 whereas the other $m - n$ are Bernoulli random variables having

$$\mathbb{P}(X_i = 1) = p = 1 - \mathbb{P}(X_i = 0), \quad i = 1, 2, \ldots, m - n. \tag{1}$$

The random variable $Y = X_1 + \cdots + X_m$ is observed, and the aim is to estimate the parameter n and provide some assessment of confidence in that estimate. In the present setting, m is the total number of mutations that are classified as either early or late, n is the number that must be early, and p is the probability that an unselected mutation is classified as early.

It is straightforward to calculate the distribution of Y. To obtain $Y = y$, the $m - n$ randomly classified mutations must result in $y - n$ being early. Hence we have

$$\mathbb{P}(Y = y) = \binom{m-n}{y-n} p^{y-n}(1-p)^{m-y}, \quad y = n, n+1, \ldots, m. \tag{2}$$

To find the maximum likelihood estimator (MLE) of n, we assume that y and p are given, so that the likelihood for the unknown parameter n is, from (2),

$$\begin{aligned} L(n) &= \binom{m-n}{y-n} p^{y-n}(1-p)^{m-y}, \quad n = 0, 1, \ldots, y; \\ &= 0, \quad n > y. \end{aligned}$$

The likelihood $L(n)$ can be maximized numerically, for example using the statistical environment R; the MLE need not be unique. Upper and lower confidence intervals for n may be found using the method described in Tingley and Li (1993). R code that implements this approach may be obtained from the authors.

Reference

Tingley M, Li C (1993) A note on obtaining confidence intervals for discrete parameters. The American Statistician 47:20-23.
